# Bond Strength of Adhesive Mortars to Substrates in ETICS—Comparison of Testing Methods

**DOI:** 10.3390/ma18214977

**Published:** 2025-10-31

**Authors:** Paweł Gaciek, Mariusz Gaczek, Paweł Krause

**Affiliations:** 1 Faculty of Civil Engineering, Silesian University of Technology, 44-100 Gliwice, Poland; 2 BOLIX S.A., 34-300 Żywiec, Poland; 3 Faculty of Civil and Transport Engineering, Poznan University of Technology, 60-965 Poznan, Poland; mariusz.gaczek@put.poznan.pl

**Keywords:** ETICS, adhesive mortars, bond strength, pull-off test, CAST method, adhesive layer thickness

## Abstract

This study investigates the bond strength of fifteen cement-based adhesive mortars used for expanded polystyrene (EPS) in External Thermal Insulation Composite Systems (ETICS). Field surveys and contractor interviews (170 questionnaires) found that adhesive layer thicknesses in real applications typically range from 15–20 mm and frequently exceed 20 mm, in contrast to the smaller values most often recommended by guidelines and technical instructions. Laboratory testing was conducted using two approaches: the standardized pull-off procedure according to EAD 040083-00-0404 (EAD and EAD′ variants) and an in-house pull-off procedure designed to reflect practical conditions of substrate type (concrete slab, silicate block), substrate orientation (horizontal, vertical), and adhesive layer thickness (10 and 20 mm). The results showed that adhesive bond strength is strongly influenced by adhesive layer thickness, substrate type, and substrate orientation. Increasing thickness from 10 mm to 20 mm on concrete substrates typically reduced bond strength by about 65–75%, while vertical orientation lowered adhesion to about half of that obtained in horizontal placement. Silicate substrates exhibited generally lower bond strength but higher variability, occasionally with ratios above unity due to their greater porosity. In some configurations, detachment occurred already during specimen preparation, underlining the variability of performance. The combined effect of increased thickness and vertical orientation on concrete substrates reduced adhesion by about 85% compared to the 10 mm horizontal baseline, highlighting the severity of unfavorable application conditions, whereas on silicate blocks, the effect was weaker but accompanied by large variability. The findings indicate that adhesive layer thickness has a stronger impact on bond strength than orientation and that substrate properties play an important role. The study provides a comparative perspective on current and alternative testing approaches, revealing significant differences in the results. The author’s testing method makes it possible to account for, in laboratory conditions, primarily the geometric shape and orientation of samples that are close to the actual form of adhesive mortar application in real insulation installations. This allows for the assessment of the properties of mortars and substrates that were not exposed under the conditions of current testing methods. The above provides a basis for further discussion on the inclusion of realistic application conditions in the evaluation of adhesive mortars used for bonding thermal insulation in ETICS, and for the validation assessment of an additional testing method, which is currently of an experimental nature.

## 1. Introduction

For many years, energy-efficient construction in Europe has been the subject of extensive legal regulations aimed at accelerating and facilitating the transition toward sustainable development [1,2]. As a result, the thermal modernization of existing buildings has gained particular importance [3,4]. At the same time, over the past several years, very dynamic growth has been observed in residential construction in Poland, both in large-scale developer projects and in single-family housing. In both cases, buildings must meet increasingly strict requirements regarding the thermal insulation of external walls [5]. The most commonly used thermal insulations for external wall systems in Poland are expanded polystyrene (EPS) and mineral wool [6,7].

Multi-family buildings constructed in Poland are usually low- and medium-rise. Data on developer projects completed in 2024 indicate that they most often have 3–5 stories [8]. In large cities, taller buildings can also be found. Most developer buildings are constructed using relatively fast technological processes, usually in monolithic reinforced concrete structures, where cement hydration and concrete curing conditions significantly affect the material properties [9]. Reinforced-concrete transverse wall systems or skeletal systems are infilled with masonry walls made of silicate, ceramic, or autoclaved aerated concrete blocks [10].

As a consequence of inaccuracies—mostly related to reinforced concrete formwork—irregularities appear in the plane of external walls [11], which in turn affect the thickness and variation in the adhesive layer for thermal insulation in ETICS. There are different methods for leveling walls before insulation [12,13]. However, these result in additional investment costs and extended construction time, which in most cases investors and contractors are not prepared for, since the standardized cost of insulation itself usually does not account for such activities—particularly in new buildings. Therefore, wall irregularities are almost always compensated for by the thickness of the adhesive layer when bonding thermal insulation in ETICS.

In the vast majority of commercial residential developments, the thermal insulation of external walls is provided by ETICS, which are the most commonly used solution both in Poland and throughout Europe [14]. The creativity of insulation system manufacturers, combined with the expectations of architects, has led to the emergence of a wide spectrum of finishing solutions that shape the architectural character of building façades. In addition to traditional thin-coat renders, proven in testing methods and in service, today’s new variants of ETICS finishes include imitations of various natural materials, such as wood, stone, as well as ceramic cladding or concrete. These may appear either as imprinted textures in a specially prepared render layer or in the form of prefabricated boards and panels bonded to the reinforced base coat, which is already covered by the draft amendment of the EAD [15]. In addition, completely new design forms have been developed, which no longer imitate other materials but constitute original solutions in themselves, e.g., the ribbed effect or the mosaic effect created from colored aggregates embedded in transparent resin. Mineral-heavy materials, such as clinker tiles or stone elements, have also been adhered to ETICS for many years, and these are likewise covered by proven testing methods [16]. Non-combustible systems and the fastening methods adapted to them for attaching insulation to walls mean that there are no restrictions on the use of ETICS with regard to building height. This significant progress in development, along with the unquestionable effectiveness in providing thermal insulation, promotes the widespread adoption of ETICS and the very large scale of implementations, further reinforced by the growth of residential construction.

Analysis of the recommended thickness of the adhesive layer for thermal insulation in ETICS between 1996 and 2024, based mainly on manuals, instructions, and guidelines, has shown that the maximum thickness of the adhesive layer for fixing insulation to walls was up to 10 mm and up to 20 mm [17]. However, it should be emphasized that the maximum thickness of up to 10 mm was the longest recommended value in general guidelines in Poland and still appears in some recommendations by adhesive manufacturers today.

In the current national guidelines [12], the maximum thickness of the adhesive layer under thermal insulation is no longer specified, while in the recommendations [13], the limit thickness is 20 mm, which at the end of 2022 replaced the long-standing recommendation of up to 10 mm. In addition, the new recommendation of increased adhesive layer thickness is conditional upon meeting the necessary requirements regarding the adhesive’s bond strength to the substrate and insulation, as well as achieving a minimum effective bonding surface of 40%. Ultimately, the thickness of the adhesive mortar is determined by the manufacturer’s guidelines, and this is a prerequisite for its application. This opens the possibility of shaping certain properties of adhesive mortars that can meet the above assumptions. It also creates the need to establish conditions and procedures for their assessment. Technical Assessments issued for insulation systems by authorized bodies usually do not specify the thickness of the adhesive layer for fixing thermal insulation, instead always providing adhesive consumption per 1 m^2^ at a relatively low level, allowing the thickness of the adhesive layer to reach, at most, a few millimeters (4–5 kg of adhesive/m^2^), assuming a minimum effective bonding surface of 40%.

In study [18], the issue of bonding mineral wool to substrates made of various boards used as sheathing in timber-frame buildings was presented, confirming the effectiveness of such bonding under specific conditions. In turn, the research work [19] presented tests on a relatively large sample of complete insulation systems, in which, among other aspects, the adhesive–substrate bond was analyzed according to the ETAG 004 method [20]. One of the many conclusions drawn was the relationship between the bending behavior of adhesive mortars and their adhesion to the substrate, also with reference to the failure patterns that occurred during sample pull-off tests. Moreover, although focused on bonded systems outside the ETICS context, cohesive zone (CZM) and concrete damage plasticity (CDP) models [21,22] show that debonding loads and failure modes are highly sensitive to the geometry of the adhesive layer and to interface properties.

It should be emphasized that the issue of adhesion of cement-based adhesive mortars, particularly to concrete, has been widely discussed and investigated by many researchers for many years. This is, of course, due to the large scale and necessity of repairing various concrete structures, as well as the need to understand the mechanisms responsible for the adhesion of repair mortars and the durability of their bond with concrete. However, there is a lack of comparable research analyses regarding the adhesion performance of adhesive mortars to substrates in ETICS under real-life conditions.

It should be remembered that wall substrates intended for thermal insulation are not limited to concrete but also include various other materials used for masonry walls. Therefore, it can be expected that, in the contact zone between the substrate and the adhesive mortar, phenomena may occur that are not necessarily consistent with those observed for concrete, and that depend on the properties of the specific substrate and the specific mortar.

The above review of the state of the art in this field, along with several important facts concerning residential construction in the context of ETICS, encourages further analysis and investigation of key insulation components in order to ensure the operational safety and durability of external wall insulation. One such relatively little-explored area is the bonding of thermal insulation to walls in ETICS.

The authors of this publication therefore decided to fill, at least partially, the gap related to research on the performance of adhesives used for bonding thermal insulation, but within the scope in which they are applied in real ETICS implementations. This concerns both the method and the geometric pattern of adhesive mortar application on the board, the thickness of the adhesive layer, the type of substrate, and the method of exposing the test specimens.

The conducted research opens up the possibility of designing specific properties and characteristics of adhesive mortars that, even with an increased thickness of the adhesive layer and varied substrates, can effectively fulfill their function in bonding thermal insulation. This contributes to improved operational safety and enhanced durability of the described insulation systems.

The proposed author’s laboratory method for testing the adhesion of adhesives to substrates provides a tool that can also be used to verify properties of mortars other than those considered so far. This enables the development of new testing methods and the enhancement of currently applied procedures for assessing the adhesion of adhesive mortars to substrates.

Taking the above into account, the following research questions were formulated: What are the most commonly encountered thicknesses of cement-based adhesive mortars in ETICS applications? How does the thickness of the adhesive layer influence the effectiveness of thermal insulation bonding? How can the maximum thickness of the adhesive layer be determined for a specific mortar and a specific substrate? To what extent do the assumptions of the adhesive testing method from EAD [15] and its requirements regarding adhesion to the substrate correspond to the real conditions of applying thermal insulation to building walls?

The research presented below is a continuation of the research concept and subject matter published in article [23], which provided the foundations and justification for the development of the authors’ own method for testing the adhesion of adhesive mortars to the substrate and insulation.

The results of the in situ and survey studies reveal the actual thickness of the adhesive layer used for bonding thermal insulation during façade insulation works. A comparison of the adhesion test results of adhesives obtained in laboratory conditions using both the standard and the author’s method has shown significant differences. For some mortars tested with the author’s method, it can be stated that they exhibit highly variable adhesion depending on the type of substrate. Therefore, the proposed additional testing method enables the evaluation of important mortar properties relevant to the practical execution of insulation works—properties that are not revealed by the standard method.

It should also be emphasized that there are mortars that, in both methods, demonstrate sufficient tensile bond strength. Furthermore, all mortars tested using the standard method meet the minimum adhesion requirements to concrete substrates under laboratory conditions. The presented new perspective on the evaluation of adhesive mortars may significantly alter the perception of their adhesion to substrates with varying characteristics and at different adhesive layer thicknesses.

## 2. Materials and Methods

All the studies were planned to form a coherent whole in relation to the research problem, taking into account both scientific and practical perspectives. The aim of the conducted research was also to provide answers to the research questions posed in the introduction.

Field observations and data obtained from the Central Statistical Office [9] made it possible to identify the materials used for wall construction and to determine the types of buildings that have predominated in the Polish multi-family housing market in recent years. This formed the basis for establishing the assumptions regarding the substrate in the adhesion tests of adhesive mortars used for bonding thermal insulation (expanded polystyrene).

Observations of actual buildings during insulation works made it possible to determine the real range of wall irregularities, providing data on the causes and quantitative characteristics of their occurrence. This directly affects the thickness of the adhesive layer between the ETICS insulation and the substrate.

A survey conducted among ETICS installers aimed to reveal the actual thicknesses of adhesive mortars used in bonding ETICS, independently of the on-site measurements.

The most common method of bonding thermal insulation boards is the ribbon-and-dab method, which features two geometric forms of adhesive application on the board. The first is the peripheral ribbon of adhesive, and the second consists of adhesive dabs applied within the inner area of the board. The laboratory testing of the adhesion of adhesive mortars to substrates using the author’s method was adapted to thicknesses similar to those occurring in real applications, taking into account one of the two geometric forms of adhesive application on the thermal insulation board—an adhesive dab in the shape of a circle.

The results of the preliminary studies served as the basis for defining the assumptions adopted in the adhesion tests performed with the author’s method.

The adhesion testing of adhesive mortars under laboratory (dry) conditions was carried out using both the author’s method and the standard method, including a variant modeled on the standard method but considering the substrates used in the author’s method. The aim was to compare the results obtained with all methods and to draw relevant conclusions.

### 2.1. Investigation of Wall Geometry and the Thickness of Adhesive Mortar for Thermal Insulation in ETICS

The examination of walls prior to insulation was carried out in a complex manner. The first element consisted of macroscopic observational studies, performed without the use of measuring tools, based solely on visual assessment. The tests concerned the surfaces of external walls of new buildings, directly from the outside environment. The assessment covered the walls of several dozen multi-family buildings intended to be fitted with ETICS insulation. Verification included the types of construction materials used, the methods of their mutual connection within a single type of material, as well as between two different wall materials. In addition to macroscopic studies, geometrical measurements of wall surface flatness were performed using straightedges of 2.0 m and 2.5 m in length. Various walls were examined, accessible mainly from stationary scaffolding, as the assessment becomes more reliable when covering the entire wall surface, not only those accessible from ground level. Wall plane deviations were examined primarily in the vertical plane, which exhibited the dominant irregularities affecting the thickness of the adhesive layer. The control straightedge was applied at wall offsets, which most often required correction of the resulting irregularities in order to maintain the plane of the external face of the insulation after bonding.

In addition, the actual thickness of the adhesive layer was examined, measured directly during the wall insulation works. The measurements were carried out using a straightedge with an accuracy of 1 mm.

### 2.2. Empirical Research—Survey-Based

In order to verify the conclusions drawn from the studies on wall geometry and the measurements of adhesive mortar thickness for bonding the thermal insulation layer to the wall substrate, a diagnostic survey was carried out among companies performing ETICS insulation works. In addition to verification, the aim was also to significantly extend the scale of the research. The survey employed telephone interviews and face-to-face conversations, in which the questionnaire served as the research tool. Individuals directly involved in the execution of ETICS insulation works were asked to assess the thickness of the adhesive layer under real construction conditions. The survey was conducted on a sample of 170 companies carrying out ETICS insulation with systems from various manufacturers operating on the Polish market. The questionnaires were not signed, and at the beginning of each interview, the respondents were informed that their answers would not entail any obligation of formal confirmation. The respondents and the companies in which they work were neither identified nor disclosed. The author of the survey concept assumed that precisely such conditions would allow for obtaining reliable information without fear of potential consequences resulting from the answers provided. The survey covered, in a cross-sectional manner, mainly micro-, small-, and medium-sized enterprises. Such companies most frequently provide insulation services in Poland, working as contractors or subcontractors. The questions were asked in different regions of Poland. The survey included several questions, of which the authors considered the following two to be relevant to the subject matter of this publication:

- What actual thickness of the adhesive layer do you most frequently encounter when applying thermal insulation in ETICS?
- What maximum thickness of the adhesive layer do you most frequently encounter when applying thermal insulation in ETICS?

The results of the survey were presented in the form of a chart.

### 2.3. Laboratory Tests

With reference to the scale of new building construction and the most commonly chosen materials for their erection, a concrete slab with specific characteristics and a silicate block used for masonry walls were selected as substrates for testing, while expanded polystyrene (EPS TR 100) was chosen as the thermal insulation. The most frequently used adhesives for bonding insulation are cement-based adhesive mortars, which are justified by cost optimization and functionality. Considering also the relatively large thickness of the adhesive layers used and the need to ensure proper performance, other types of known adhesives for ETICS would have neither economic nor practical justification. Covering the, as can be seen, significant wall irregularities also results in relatively high consumption of adhesives for bonding thermal insulation.

To assess the adhesion of adhesive mortars to the substrate, fifteen cement-based mortars for bonding EPS were selected. Each mortar is part of an insulation system that has been issued a Technical Assessment. It was therefore assumed that all mortars should meet the requirements of the EAD [15] regarding adhesion to the substrate.

All mortar tests were performed exclusively under laboratory conditions. Two methods were used to test the adhesion of adhesives to the substrate. The first was the method specified in EAD [15], from which an additional variant was derived, referred to in this article as the “EAD-based method,” due to the lack of consistency of the substrate with the reference document. The second was an original method called the Central Adhesive Spot Test (CAST) [24]. The fundamental difference between the methods concerns the form of sample preparation, while their similarity lies in the planar shape of the detached element (a square block with 50 mm sides) and the pull-off technique. Both methods also share the curing conditions related to temperature and air humidity—laboratory conditions consistent with EAD [15].

In the tests, the bulk densities of all mortars used were determined. These values are not provided in the article so as not to enable the identification of individual adhesive mortar manufacturers, for whom such values may be distinctive.

#### 2.3.1. Laboratory Testing of Adhesive Mortars by Means of the CAST Method

The CAST method is used to assess the adhesion of an adhesive mortar dab to the substrate and thermal insulation by pulling off its central part. In this study, the method was applied to evaluate the adhesion of the adhesive to the substrate. The test was designed in such a way that the specimen would reflect, to the greatest possible extent, the real conditions of bonding thermal insulation. This is related both to the shape of the specimen and the method of its bonding, the type of substrate, as well as the orientation of the specimen in relation to horizontal and vertical planes during bonding and curing. The specimens were also bonded under pressure similar to that applied when bonding thermal insulation boards to walls in actual ETICS applications. The method requires the adhesion of the adhesive mortar to be tested on each type of substrate to which the adhesive mortar is intended to be applied.

In this method, the test specimen is an adhesive dab of appropriate mass or volume and defined thickness, placed in its most advantageous form on a square EPS TR 100 plate, the size of which is matched to the dab. The measured quantity of adhesive mortar is applied with a working tool onto a horizontally placed EPS plate with dimensions of 15 cm × 15 cm × 5 cm, positioned on a scale. The placement of the specimen on the scale during adhesive application facilitates control of the mortar amount relative to the intended mass. Two adhesive dab thicknesses were adopted for the tests, namely 10 mm and 20 mm. A 10 mm dab was formed from 200 g of fresh adhesive mortar, while a 20 mm dab was formed proportionally from 400 g of the same mortar. Each of the fifteen adhesive mortars was prepared in accordance with the instructions provided on the packaging. Potable water was used for mixing in the quantity specified by the manufacturer. In cases where a range of water content was indicated, the mid-value of the recommended range was adopted.

After preparation, the specimens were bonded to the substrate in horizontal (5 specimens) and vertical (5 specimens) positions. The thickness of the joint was adjusted using spacers to ensure repeatability of its dimensions. EPS specimens bonded vertically were prepared in such a way that the EPS plate with the adhesive dab was supported from below, analogously to the support provided for thermal insulation boards bonded to walls in actual ETICS applications. This prevented the specimens from shifting during testing under their own weight.

Curing of the specimens was carried out under conditions specified in EAD 040083-00-0404 [15]. Specimens bonded horizontally were cured horizontally, and specimens bonded vertically were cured vertically, in the same place where they had been bonded, without being moved. After the curing period, the EPS was mechanically removed from the surface of the adhesive dabs, and their surfaces were then carefully cleaned of residual mortar. In the next step, square elements with 50 mm sides were cut from the central part of the application. The cuts extended approximately 2 mm into the substrate. A diamond blade was used to minimize vibration during the cutting of the cured mortar layer. A steel plate with a connector to the pull-off device was then bonded with fast-setting adhesive to the square. All specimens were pulled off in a horizontal position, including those bonded and cured vertically.

Two types of substrates were used in the tests:

- Smooth concrete slabs, tensile strength (perpendicular pull-off) above 1.8 MPa, density approx. 2247 kg/m^3^, water absorption 28.1 g/(m^2^ × s^0.5^), test according to EN 772-11:2011 [25];
- Silicate blocks intended for masonry walls, density 1410–1600 kg/m^3^, water absorption 188.6 g/(m^2^ × s^0.5^), test according to EN 772-11:2011 [25].

These substrates were coded as s1 and s2.

Five replicate pull-off tests were performed for each configuration. The tests were carried out for each adhesive (a1–a15) and each substrate (s1, s2), for specimens bonded in the horizontal (o1) and vertical (o2) orientations, and for adhesive layer thicknesses of 10 mm (t1) and 20 mm (t2).

For each adhesive, this resulted in 8 combinations of substrate–orientation–thickness, referred to as “conditions”, which were coded as c1–c8 and are summarized in Table 1.

**Figure 1:**
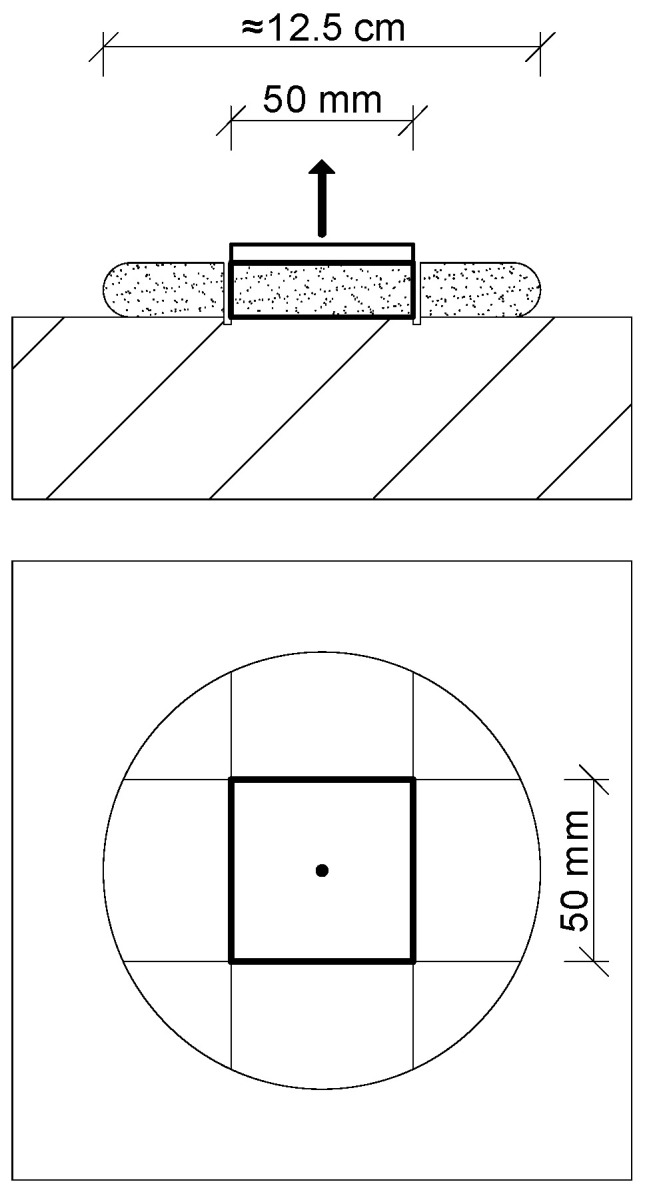
Layout of the 50 mm × 50 mm square section cut from a round adhesive dab and pulled off from the concrete substrate (CAST method).

**Figure 2:**
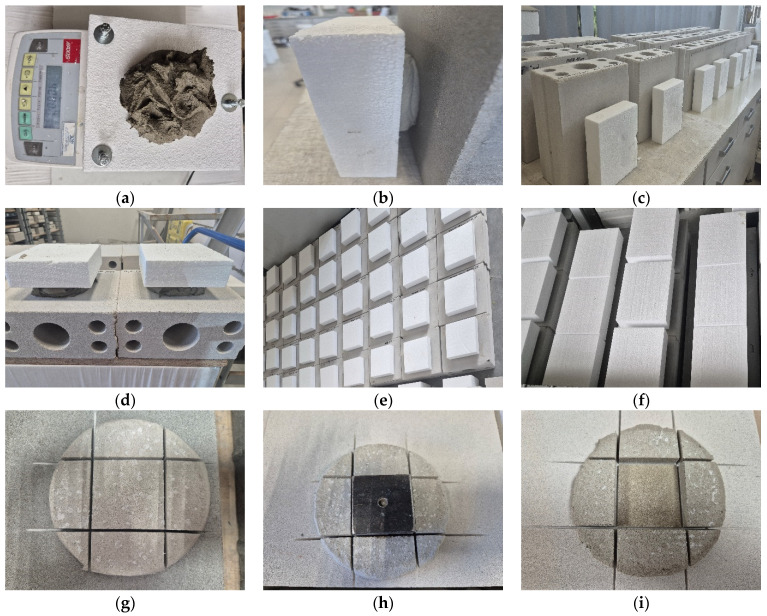
Example images showing the process of specimen preparation, curing, and pull-off: (**a**) adhesive dab of fresh mortar on an EPS plate with spacers determining the thickness of the dab after bonding the specimen to the substrate, specimen placed on a scale; (**b**) specimen bonded and cured in a vertical position, substrate: concrete slab; (**c**) specimens bonded and cured in a vertical position, substrate: silicate blocks; (**d**) specimens bonded and cured in a horizontal position, substrate: silicate block; (**e**,**f**) view of specimens cured in a horizontal position; (**g**) adhesive dab after removal of EPS, specimen with the central element cut out; (**h**) steel block bonded to the central element of the specimen, serving as a connector to the pull-off device; (**i**) view of specimen after detachment of the central element using the pull-off device.

A total of 40 test specimens were prepared for each adhesive mortar. Figure 1 shows the diagram of the test specimen, and Figure 2 presents step by step the main elements of the specimen preparation and pull-off process.

**Figure 3:**
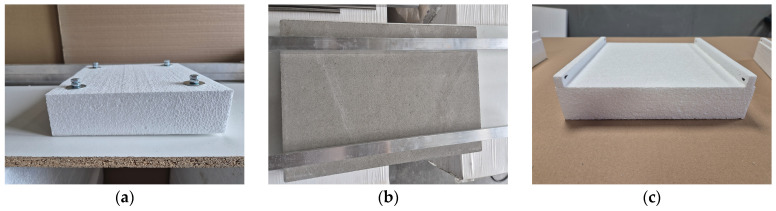
Examples of spacer elements determining the thickness of the adhesive dab on the EPS board: (**a**) steel pins embedded in the EPS plate; (**b**) aluminum guides with a square cross-section placed on the concrete slab; (**c**) EPS strips bonded to the EPS plate.

Additionally, Figure 3 shows examples of spacer elements used to achieve the required thickness of the adhesive dab on the EPS board.

#### 2.3.2. Laboratory Testing of Adhesive Mortars by the Standard Method and the Method Based on EAD [15]

The test was carried out under laboratory conditions. Three types of substrates were used: two defined as in Section 2.3.1, and additionally, the substrate specified in EAD [15], represented by a SOLONA-type board. Since two of the three substrates had a different specification than that defined in the EAD [15], these parts of the study were referred to as “EAD-based” and are denoted as EAD′.

Each of the 15 adhesive mortars was prepared in accordance with the manufacturer’s instructions. The mortars were mixed with the amount of water recommended on the packaging. In cases where a range of water content was specified, the mid-value of the recommended range was adopted.

On each of the three substrates, the prepared mortars were applied in a thickness of 4 mm using so-called guides. The specimens were produced and cured in a horizontal position. After the curing period, square elements with 50 mm sides were cut from the specimens, with incisions reaching about 2 mm into the substrate, in accordance with the EAD [15] recommendations. A steel plate with a connector to the pull-off device was bonded to the separated square using fast-setting adhesive. All specimens were pulled off in a horizontal position.

**Figure 4:**
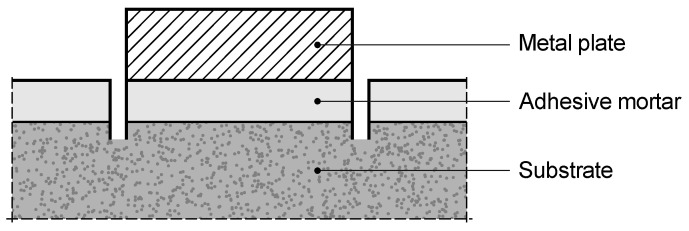
Test specimen layout for pull-off testing [15].

Five test specimens were prepared for each combination of substrate and mortar. The test procedure (pull-off test) is illustrated in Figure 4, based on [15].

The substrates used in the study were:

- “Normative” concrete slabs of the Solana type, density approx. 2363 kg/m^3^, water absorption 25.4 g/(m^2^ × s^0.5^), test according to EN 772-11:2011 [25]—used exclusively for the method specified in EAD 040083-00-0404 [15], laboratory conditions, adhesive layer thickness 4 mm.
- Concrete slabs as defined in Section 2.3.1.
- Silicate blocks as defined in Section 2.3.1.
- These substrates were coded as s0, s1, and s2, respectively.

### 2.4. Empirical Data Analysis

The empirical data consisted of bond strength values obtained from pull-off tests, reflecting the adhesion between the adhesive mortar and the substrate.

#### 2.4.1. Distributional Assessment

The first step in the analysis was to evaluate the distributional form of the empirical data, in order to select appropriate statistical tools. Since each test series consisted of only five replicates, conventional normality tests such as Shapiro–Wilk (SW) or Kolmogorov–Smirnov (KS) were not considered reliable due to their low statistical power with small sample sizes. Instead, distributional fit was evaluated using three general goodness-of-fit statistics—Kolmogorov–Smirnov (KS), Cramér–von Mises (CvM), and Anderson–Darling (AD)—together with two information criteria: the Akaike Information Criterion (AIC) and, as the primary selection metric, the Bayesian Information Criterion (BIC). Quantile–quantile (Q–Q) plots were also visually inspected for each dataset. Four candidate probability distributions were considered: Weibull, normal, log-normal, and gamma. All data analyses were performed in R [26] within the RStudio IDE [27], using the fitdistrplus [28], MASS [29], and survival [30,31] packages.

#### 2.4.2. Reporting of Fit Measures

Formal goodness-of-fit statistics (KS, CvM, AD) and information criteria (AIC, BIC) were reported as point estimates only. Given the small sample size (*n* = 5), *p*-values were not considered informative and could obscure the clarity of the results. Model comparisons were instead based on direct inspection of BIC differences and visual evaluation of Q–Q plots. In several cases, BIC suggested that the normal distribution provided the best fit, whereas Q–Q plots revealed systematic deviations in the tails. For this reason, both numerical and visual assessments were considered jointly, and descriptive as well as inferential procedures were selected with preference for methods minimally sensitive to distributional assumptions. Distributional parameters were estimated by maximum likelihood (MLE).

#### 2.4.3. Descriptive Statistics

Both classical and robust descriptive measures were reported. Classical measures included arithmetic mean, median, standard deviation (SD), standard error of the mean (SE), variance, coefficient of variation (CV), and interquartile range (IQR). Robust alternatives included the median absolute deviation (MAD), the mean absolute deviation from the median (MedAD), and the scaled median absolute deviation (MedAD_n_ = 1.4826 × MedAD), which serves as a robust estimator of the standard deviation under the assumption of normality.

#### 2.4.4. Bootstrap Resampling

To address uncertainty due to small sample sizes, bias-corrected and accelerated (BCa) 95% confidence intervals were estimated for both mean and median using 10,000 bootstrap replications (implemented in R [26] using the boot package [32,33]). Bootstrap-based point estimates were reported together with their corresponding confidence limits.

#### 2.4.5. Comparative Metrics

In accordance with EAD 040083-00-0404 [15], the arithmetic mean of five pull-off tests was retained as the primary comparative metric of bond strength. This approach was applied in the conventional method (EAD), which follows the provisions of the European Assessment Document and requires testing on the normative concrete substrate. The same procedure was also applied in the EAD′ variant, which is analogous to EAD but performed on alternative substrates (non-normative concrete slabs and silicate blocks). In the in-house method (CAST, Central Adhesive Spot Test), descriptive statistics were additionally extended to include the bond-strength ratio for 20 mm vs. 10 mm mortar thickness (t2/t1), the ratio between vertical and horizontal orientations (o2/o1), and compound ratios combining thickness and orientation (c4/c1, c8/c5), accompanied by bootstrap confidence intervals. Importantly, these ratios are not computed on individual pulls but on aggregated statistics: first, replicate results within each adhesive and case are averaged to obtain a per-adhesive mean; next, these per-adhesive means are aggregated across adhesives to obtain the grand mean and the median of means for each case. Unless specified, all reported quantities are based on per-adhesive mean bond strength.

#### 2.4.6. Correlation Analysis

In the EAD/EAD′ method, the relationship between the per-adhesive mean bond strength and the width of its 95% CI (BCa) was assessed separately for each substrate to gauge how substrate choice affects estimation uncertainty. The primary measure was the biweight midcorrelation (bicor) (WGCNA package [34,35]), an outlier-robust estimator based on Tukey’s biweight; bootstrap 95% CIs for bicor were obtained. For robustness, rank-based monotonic associations were also reported using Spearman’s *ρ* and Kendall’s *τ* (with exact *p*-values). Correlation magnitudes were interpreted alongside MedAD_n_ and IQR computed across per-adhesive means to contextualize cross-adhesive dispersion.

#### 2.4.7. Inferential Statistics

For the in-house method CAST (Central Adhesive Spot Test), the primary inferential framework was a factorial (fixed-effects) design with three factors: substrate (concrete slab/silicate block), orientation (horizontal/vertical), and adhesive layer thickness (10 mm/20 mm). PERMANOVA (vegan package [36]) was used to partition variance across main effects and interactions, reporting R^2^ and permutation *p*-values for each effect. Where helpful, results were complemented by permutation ANOVA (lmPerm package [37]) to obtain eta-squared (*η*^2^) per effect, and by epsilon-squared (*ε*^2^) computed from rank-based summaries (effectsize package [38]). For interpretability, nonparametric post hoc contrasts were run for each main factor using pairwise Wilcoxon rank-sum tests with Holm adjustment; pairwise effect sizes were expressed as Cliff’s *δ* and Vargha–Delaney A (effsize package [39]), with bootstrap confidence intervals (boot package [32,33]). As a robustness check against non-additivity, factorial effects were verified using the aligned rank transform (ARTool package [40,41]). A global Kruskal–Wallis test across the eight combined cases (s × o × t; c1–c8) was also computed for CAST as a compact omnibus check, reporting epsilon-squared (*ε*^2^) as the effect size (effectsize package [38]).

In addition to the factorial design, a one-way CAST analysis was performed across the eight combined conditions (c1–c8), with a single eight-level factor as the independent variable. A similar omnibus framework to that used for EAD was applied: Kruskal–Wallis (effect size *ε*^2^ with bootstrap CIs; boot) and PERMANOVA (R^2^ and permutation *p*; vegan package), and, where informative, permutation ANOVA to obtain *η*^2^ (lmPerm package). When omnibus tests were significant, pairwise Wilcoxon rank-sum comparisons with Holm correction were explored, with pairwise effect sizes summarized by Cliff’s *δ* and Vargha–Delaney A (effsize package) and bootstrap CIs (boot package).

For the conventional method (EAD), the independent variable was substrate (three levels). Overall group differences were assessed with the Kruskal–Wallis test (effect size *ε*^2^ with bootstrap CIs; boot package [32,33]). In parallel, the approximate K-sample Fisher–Pitman permutation test (coin package [42,43]) and permutation ANOVA (lmPerm package [37]) were applied to obtain *η*^2^. PERMANOVA (vegan package [36]) was used as an additional nonparametric validation of omnibus differences, reporting R^2^ and permutation *p*-values. When omnibus tests were significant, pairwise Wilcoxon rank-sum comparisons with Holm correction were performed, and pairwise effect sizes were summarized using Cliff’s *δ* and Vargha–Delaney A (effsize package [39]) with bootstrap CIs (boot package [32,33]).

Unless stated otherwise, omnibus effect sizes are reported as PERMANOVA R^2^ and global Kruskal–Wallis *ε*^2^, whereas pairwise effect sizes are summarized by Cliff’s *δ* and Vargha–Delaney A (VD-A).

All descriptive, bootstrap, and inferential analyses were implemented in R [24], using the following packages: fitdistrplus [28], MASS [29], survival [30,31], boot [32,33], WGCNA [34,35], vegan [36], lmPerm [37], effectsize [38], effsize [39], ARTool [40,41], coin [42,43], lsr [44], and dplyr [45].

The R scripts used for descriptive, bootstrap, and inferential analyses are available from the corresponding author.

The optimization of R scripts and language editing was supported by generative artificial intelligence (GenAI) tools.

## 3. Results and Discussion

### 3.1. Wall Adhesive Mortar for Thermal Insulation in ETICS

**Figure 5:**
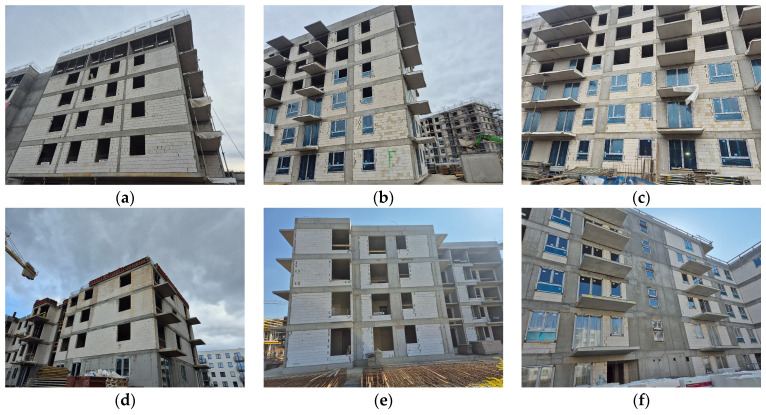
Examples of buildings under construction prior to thermal insulation: (**a**–**f**) reinforced concrete and silicate block walls.

As a result of the observations carried out on construction sites, it was established that the materials most commonly used at present for the construction of walls in low- and medium-rise developer buildings are reinforced concrete and silicate blocks. Typical structures of developer buildings, most frequently observed on construction sites in large cities, are shown in Figure 5.

**Figure 6:**
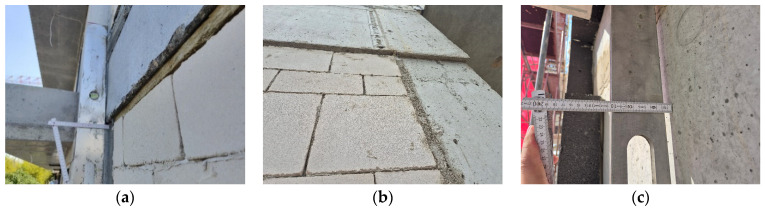

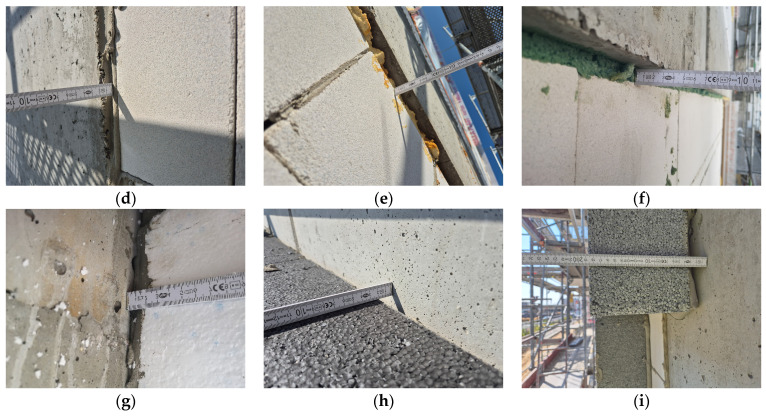
Examples of wall irregularities caused by the lack of optimal alignment of reinforced concrete formwork, as well as reinforced concrete combined with infill masonry of silicate blocks, and the resulting thicknesses of the cement-based adhesive mortar layer for thermal insulation: (**a**) maximum curvature of 20 mm estimated with a 2 m straightedge and measuring gauge, resulting from protrusion of the reinforced concrete tie beam; (**b**) offset in the reinforced concrete structure caused by formwork displacement; (**c**) maximum wall curvature of 17 mm, resulting from the curvature of the formwork used for reinforced concrete, estimated with a 2.5 m straightedge and measuring gauge; (**d**) offset of 16 mm between reinforced concrete and the silicate block wall, measured vertically with a measuring gauge; (**e**) horizontal offset of 29 mm between the reinforced concrete tie beam and the silicate block wall, measured with a measuring gauge; (**f**) horizontal offset of 31 mm between the reinforced concrete tie beam and the silicate block wall, measured with a measuring gauge; (**g**) adhesive layer thickness of 14 mm; (**h**) adhesive layer thickness of 20 mm; (**i**) adhesive layer thickness of 33 mm.

Despite the relatively high dimensional accuracy of the silicate blocks themselves, wall irregularities in many buildings constructed with combined technologies—namely reinforced concrete frames cast in formwork with infills of wall blocks—generate substrate unevenness, which in turn results in the formation of adhesive joints for bonding thermal insulation in ETICS within the range most commonly of 10–25 mm. Some causes of these irregularities and their measurement are shown in Figure 6.

### 3.2. Results of the Survey Concerning the Assessment of the Actual Thickness of the Adhesive Layer for Thermal Insulation

As a result of the survey, information was obtained that corresponds with the findings of observations and measurements carried out on construction sites.

To the question: “What actual thickness of the adhesive layer do you most frequently encounter when applying thermal insulation in ETICS?” almost 37% of respondents answered 15 mm, slightly fewer (35.7%) indicated that they most often encounter an adhesive layer thickness of 20 mm. Just over 20% of respondents indicated 10 mm as the thickness most frequently occurring in their projects, while slightly more than 8% stated that the standard adhesive layer during bonding of thermal insulation exceeds even 20 mm.

**Figure 7:**
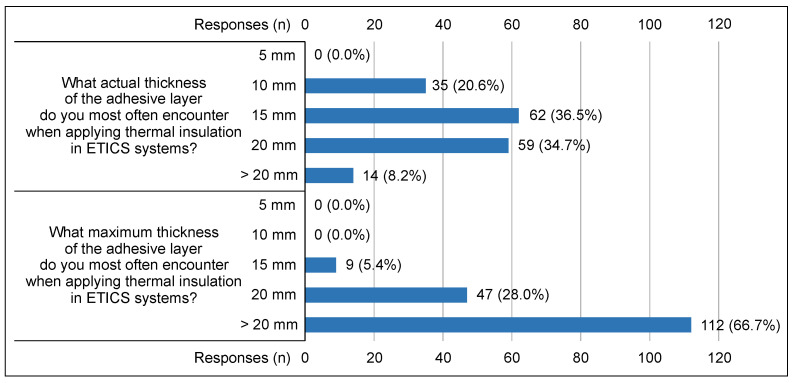
Partial results of the survey on the thickness of the adhesive layer for thermal insulation.

To the next question: “What maximum thickness of the adhesive layer do you most frequently encounter when applying thermal insulation in ETICS?” nearly 67% of respondents answered “above 20 mm”; 28% considered a 20 mm adhesive layer as the maximum most frequently used, and only just over 5% indicated 15 mm as such a layer. Partial results of the survey, including the indicated questions and the responses provided, are presented in Figure 7.

### 3.3. Bond Strength Results and Analysis

#### 3.3.1. Descriptive Statistics

1. Distributional Characteristics of the Data

Prior to computing descriptive statistics, the distributional properties of each test series were assessed to inform the selection of appropriate summary measures. Out of 153 test series (45 from the conventional method, EAD/EAD′, and 108 from the in-house method, CAST), the normal distribution was selected by the Bayesian Information Criterion (BIC) in only four cases. In contrast, the log-normal, Weibull, and gamma distributions were selected 71, 68, and 10 times, respectively. Visual inspection of quantile–quantile (Q–Q) plots further confirmed the generally poor fit of the empirical data to the normal distribution. These findings justified the use of robust descriptive statistics, such as the median, interquartile range (IQR), mean absolute deviation (MAD), scaled median absolute deviation (MedAD_n_), and bootstrap confidence intervals (BCa 95% CI), all of which are less sensitive to deviations from normality.

**Figure 8:**
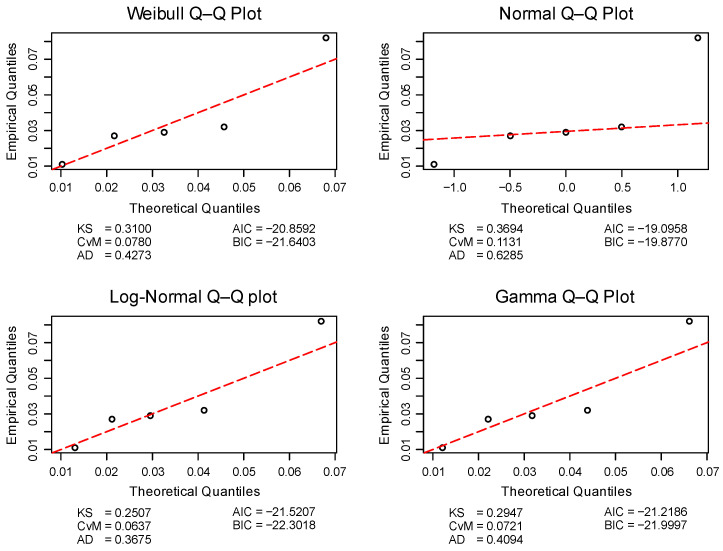
Quantile–quantile (Q–Q) plots of four probability distributions (Weibull, normal, log-normal, gamma) fitted to bond strength data for adhesive a1 under conditions c4.

Figure 8 illustrates distributional fitting for adhesive a1 under conditions c4. The upper row presents the Q–Q plots for the Weibull distribution (left) and the normal distribution (right), while the lower row contains the log-normal and gamma cases. The Q–Q plot for the normal distribution exhibits clear deviations of the empirical quantiles from the theoretical line in both tails, indicating a poor fit. This visual impression is consistent with the information criterion values, where the normal model shows the poorest BIC (−19.88). By contrast, the log-normal distribution (BIC = −22.30) provides the best overall fit, with Weibull and gamma also outperforming the normal distribution. This example reflects the general pattern observed across the dataset and justifies the use of robust descriptive measures and nonparametric inferential methods.

2. Results for the CAST Method

**Figure 9:**
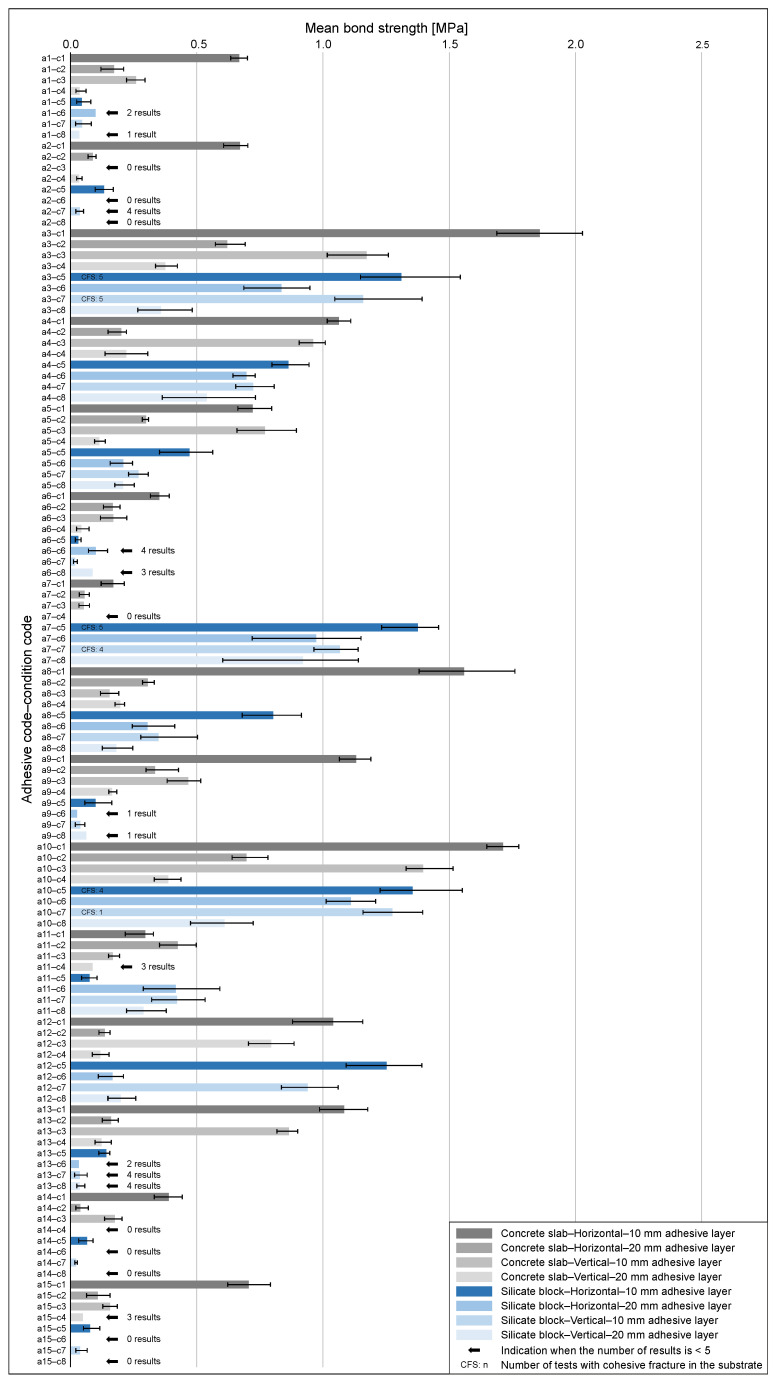
CAST method—Mean bond strength with 95% bootstrap confidence intervals for adhesives a1–a15 under conditions c1–c8.

For the CAST method, the results of bond strength measurements are summarized in Figure 9, which presents the mean values with 95% bootstrap confidence intervals for all adhesives (a1–a15) under eight CAST conditions (c1–c8). For the concrete substrate (c1–c4), the highest bond strength was obtained for the horizontal orientation with a 10 mm adhesive layer (c1), with a median of about 0.72 MPa and individual maxima approaching 1.9 MPa. Increasing the thickness to 20 mm and/or switching to vertical orientation (c2–c4) led to a substantial reduction, with medians between 0.12 and 0.36 MPa and minima close to zero. For the silicate block substrate (c5–c8), the medians were generally lower, in the range of 0.14–0.27 MPa (≈0.30 MPa when results based on a single measurement were excluded), but the data showed wide scatter, including occasional maxima above 1.3 MPa.

Vertical orientation (c7–c8) again resulted in low medians and broad variability, and several configurations provided fewer than five valid results. These findings confirm the strong influence of both substrate type and test configuration: thin horizontal layers on concrete consistently performed best, whereas increased thickness and vertical orientation systematically reduced adhesion, and silicate blocks exhibited lower typical values combined with higher scatter.

The distribution of minimum, maximum, and median bond strength values for each condition (c1–c8) is summarized in Table 2. This table provides a concise overview of the typical and extreme adhesion levels observed under the CAST method.

Robust (i.e., outlier-resistant) dispersion indicators (MedAD_n_ and IQR) confirm that the concrete—horizontal orientation—10 mm adhesive layer case (c1) is the most stable configuration. Departures from this reference—greater adhesive layer thickness and vertical orientation, especially on silicate substrates—are associated with wider within-configuration scatter.

In Figure 9, additional annotations are provided to clarify cases where fewer than five valid results could be obtained. Such situations typically occurred when very low adhesion caused detachment of specimens already during preparation; the exact number of valid results is indicated next to the respective bars. Furthermore, the notation “CFS: n” denotes the number of tests in which cohesive fracture occurred within the substrate, if such a situation was observed.

**Figure 10:**
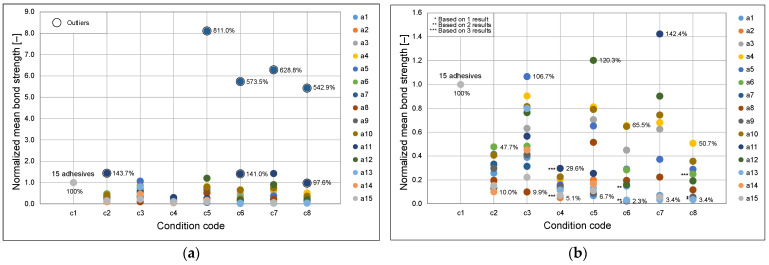
CAST method—Normalized mean bond strength for adhesives a1–a15 under conditions c1–c8: (**a**) all data; (**b**) data excluding outliers (>1.5 IQR).

Figure 10 shows the normalized mean bond strength values for adhesives a1–a15 under CAST conditions c1–c8, expressed relative to the reference configuration (c1: concrete slab–horizontal–10 mm adhesive thickness). Panel (a) includes all data, whereas panel (b) excludes outliers defined as values exceeding 1.5 times the interquartile range (IQR).

The results demonstrate that, with very few exceptions, all other conditions yielded reduced adhesion compared to c1. Only three cases without outliers, and nine cases when outliers were included, exhibited higher normalized values. The most pronounced decrease occurred for c4 (concrete slab–vertical–20 mm), with bond strength reduced by approximately 70–95%. Silicate block substrates (c5–c8) generally showed lower minimal bond strength but greater variability, ranging from low to high values. The widest spreads were observed for conditions c7 and c5. This substrate also exhibited the highest number of outliers, although these were limited to only two adhesives, with a single case being markedly deviating. For the concrete substrate, the widest spreads were observed under condition c3. These findings highlight the dominant role of adhesive thickness and substrate orientation in reducing bond strength when compared to the thin horizontal concrete reference. In Figure 10b, additional markers indicate cases where the mean was calculated from fewer than five valid results: a single asterisk * denotes means based on one result, double asterisks ** on two results, and triple asterisks *** on three results. For cases with four valid results (four instances in total), the means were considered approximately equivalent to those obtained from five results and were therefore not specifically marked.

In continuation of our previous work [23], where the influence of adhesive layer thickness was highlighted, the present study provides a much broader comparison of bond strength ratios between 20 mm and 10 mm mortar layers (t2/t1). This comparison was carried out for both substrates (concrete slab and silicate block) and orientations (horizontal and vertical). The corresponding mean and median values, together with 95% bootstrap confidence intervals, are summarized in Table 3.

**Figure 11:**
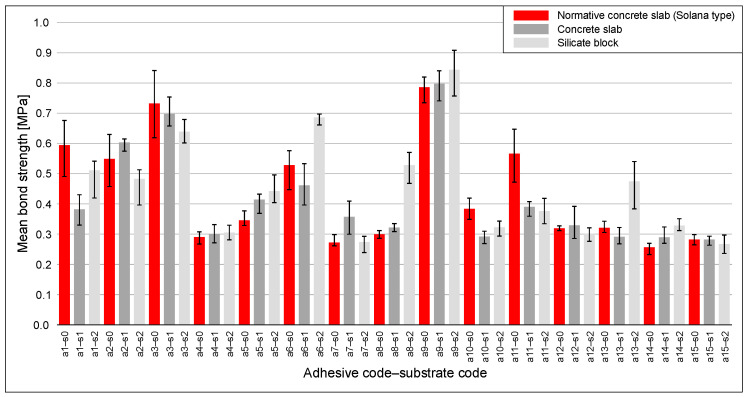
EAD/EAD′ methods—Mean bond strength with 95% bootstrap confidence intervals for adhesives a1–a15 across three substrates.

Two datasets were analyzed: ALL, which includes all test results, and LE1, which includes only results where the bond strength ratio is less than or equal to 1. The results indicate that both the change in mortar layer thickness and the type of substrate had a significant impact on the measured bond strength ratios. For the concrete slab (s1), the bond strength ratio remained consistently below 0.35, suggesting a substantial reduction in adhesion when the layer thickness increased, typically exceeding 65–75%. In contrast, the silicate block substrate (s2) exhibited considerably higher ratios, with several values exceeding 1.0 in the unfiltered dataset. This implies that, in many cases, thicker mortar layers achieved better adhesion than thinner ones. Even after restricting the dataset to ratios ≤ 1, the silicate block consistently outperformed concrete in terms of bond strength. In the full dataset, the median values are noticeably lower than the means, confirming the presence of several high outliers and indicating a skewed distribution. This behavior is likely related to the higher porosity of silicate blocks (typically 0.27–0.35 m^3^/m^3^ compared to 0.15–0.22 m^3^/m^3^ for concrete [46]), which—provided that the mortar mix contains appropriate additives to retain water—facilitates deeper penetration of hydration products and stronger mechanical–chemical anchorage within the pore structure [47,48]. It should be noted, however, that the favorable t2/t1 ratios obtained for the silicate block do not imply higher absolute bond strength values. As shown in Figure 10 and Figure 11, the silicate block conditions were generally associated with lower bond strength and greater variability than concrete. The relative comparison indicates that increasing the adhesive thickness caused a much stronger reduction in adhesion on the concrete substrate than on the silicate block, which explains the relatively higher t2/t1 ratios for the latter.

Following the approach used for thickness ratio (t2/t1), the bond strength ratio between vertical and horizontal orientations (o2/o1) was evaluated. Table 4 reports the mean and median values, accompanied by 95% bootstrap confidence intervals.

The systematically lower mean bond strengths observed for substrates in vertical orientation may be associated with two main mechanisms. First, reduced and less uniform contact pressure during application against a vertical face limits initial contact, wetting, and compaction of the mortar at the interface. Furthermore, unlike in horizontal orientation, the adhesive mortar in vertical orientation is not subjected to the minor but systematic pressure from its own weight and that of the insulation board. Second, gravity-driven redistribution of mixing water during early curing may create local gradients in water availability at the adhesive–substrate interface, potentially disturbing the fresh interfacial zone and being accompanied by slight sagging or micro-slippage of the mortar. These effects can hinder cement hydration locally, reduce micro-mechanical interlocking, and promote uneven setting and hardening, which may introduce additional stresses detrimental to adhesion. Importantly, the influence of these processes depends on the substrate: on low-suction surfaces such as concrete, limited capillary absorption exacerbates downward water migration and instability, whereas on more absorptive substrates such as silicate blocks, capillary suction helps retain water near the interface and promotes early interlock. The bond strength of a given adhesive mortar under these conditions depends on its composition.

These mechanistic considerations are consistent with the statistical results presented in Table 4. For the concrete substrate (s1), the o2/o1 ratios cluster around 0.5, indicating that adhesion in vertical orientation is about half that in horizontal orientation. For the silicate block (s2), the effect is less pronounced, and confidence intervals are wider, reflecting higher variability across adhesives. This suggests that the substrate’s pore structure and water absorption capacity may modulate the extent to which vertical orientation reduces bond strength.

When analyzed using the LE1 dataset, orientation effects (o2/o1) proved weaker than thickness effects (t2/t1). On concrete substrates, increasing thickness reduced adhesion to about one quarter of the reference (t2/t1 ≈ 0.25), while vertical orientation preserved about one half (o2/o1 ≈ 0.50). On silicate blocks, thicker layers retained about 50–60% of the reference adhesion (t2/t1), and vertical orientation retained about 60–70% (o2/o1). Thus, while both factors significantly reduced bond strength, thickness exerted the stronger effect, particularly on concrete.

For a compact check that jointly reflects thickness and orientation, Table 5 reports the ratios c4/c1 (concrete: vertical 20 mm vs. horizontal 10 mm) and c8/c5 (silicate: vertical 20 mm vs. horizontal 10 mm), with means, medians and BCa 95% CIs for the full set (ALL) and the truncated set (LE1: ratios ≤ 1).

These compound ratios are consistent with the trends above: on concrete, moving simultaneously from (o1, t1) to (o2, t2) typically reduces adhesion more markedly than on silicate, where the distribution is highly skewed due to cases with ratios > 1.

The experimental trends observed for layer thickness and substrate orientation are also consistent with computational findings reported in the literature. Cohesive zone (CZM) and concrete damage plasticity (CDP) models indicate that debonding loads and failure initiation are highly sensitive to the geometry, thermomechanical behavior, and boundary conditions of the adhesive interface. Numerical simulations of bonded systems [21,22] show that increasing adhesive layer thickness or bond length produces steeper interfacial stress gradients and localized damage zones, promoting earlier debonding—a mechanism consistent with the reduction in bond strength observed in the present study.

3. Results from the EAD and EAD′ methods

Figure 11 reports mean bond strength with 95% BCa confidence intervals for adhesives a1–a15 on the three EAD/EAD′ substrates: normative concrete slab (Solana type) (s0), concrete slab (s1), and silicate block (s2).

Aggregating across adhesives, the ordering by grand means was s2 ≥ s0 ≥ s1 (≈0.452, 0.435, 0.414). At the per-adhesive level, the highest mean occurred on s2 in 7/15 cases, on s0 in 5/15, and on s1 in 3/15. Between-substrate differences were often modest, as CIs frequently overlapped; clear (non-overlapping) advantages appeared in 5/15 cases for s2 > s0, 3/15 for s2 > s1, and 3/15 for s0 > s1, while for several adhesives, all three CIs overlapped. Robust cross-adhesive dispersion was largest on s2 and smallest on s1 (MedAD_n_ ≈ 0.178, 0.109, 0.097; IQR ≈ 0.206, 0.263, 0.142, for s2, s0, s1, respectively). To relate level and uncertainty, bicor between the per-adhesive mean and its CI width was computed per substrate: the association was strongest on s0 (bicor ≈ 0.91) and moderate on s1 and s2 (≈0.42 and 0.53). In practical terms, on the normative substrate, higher mean levels tend to be accompanied by disproportionately wider CIs, indicating greater estimation uncertainty exactly where the mean is higher; on the other two substrates, the tendency is present but markedly weaker. Rank-based checks were consistent: on s0, Spearman’s *ρ* ≈ 0.83 (*p* < 0.001) and Kendall’s *τ* ≈ 0.67 (*p* = 0.001) confirmed a strong positive association, whereas on s1 and s2 the associations were only moderate and not significant at *n* = 15 (Spearman’s *ρ* ≈ 0.43/0.44; Kendall’s *τ* ≈ 0.28/0.30).

Under the EAD/EAD′ framework, no zero-adhesion results (below the measurable detection threshold) were observed in 225 pull-off tests (15 adhesives × 3 substrates × 5 replicates). By contrast, in CAST, such cases occurred on both substrates. On concrete, zero adhesion was observed for five adhesives and spanned two distinct condition types (orientation × thickness), with replicate patterns 5/5, 5/5, 2/5, 5/5, 2/5 (in total 19/25 zero pulls). On silicate, zero adhesion was observed for seven adhesives and spanned three distinct condition types, with replicate patterns 3/5, 5/5, 1/5, 4/5, 3/5, 5/5, 5/5, 1/5, 1/5, 4/5, 5/5, 2/5, 4/5, 1/5, 5/5, 5/5 (in total 54/80 zero pulls). Here, a notation such as “3/5” denotes 3 zero results out of 5 tests in a given combination of adhesive and condition (substrate–orientation–thickness). This contrast indicates a potential safety concern: the standardized EAD/EAD′ setup did not surface very low adhesion, whereas CAST revealed multiple below-threshold cases under realistic use-case combinations.

**Figure 12:**
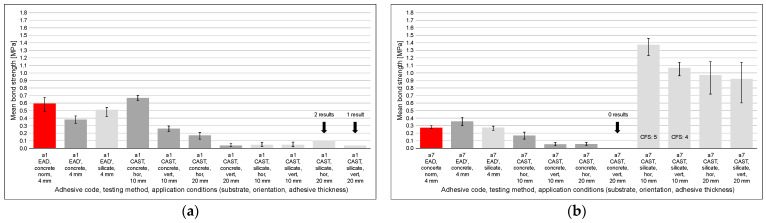

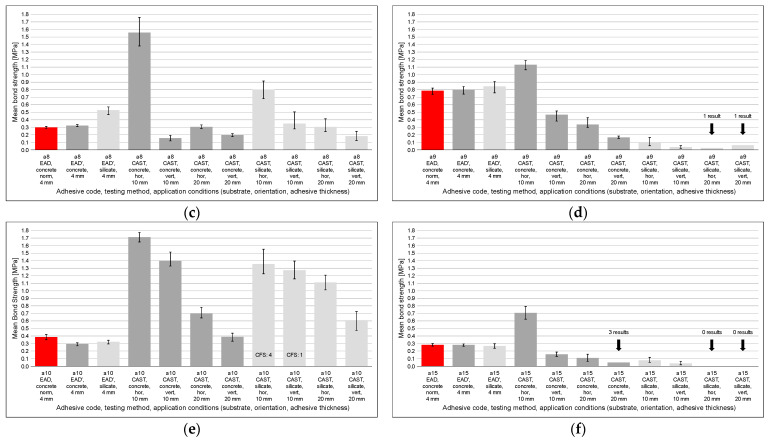
Comparison of mean bond strength from the EAD/EAD′ and CAST methods, for example, adhesive mortars: (**a**–**f**) adhesives 1, 7, 8, 9, 10, and 15. Error bars: 95% BCa bootstrap confidence intervals (CI). Arrows indicate cases with *n* < 5 (incomplete set of test results). CFS: *n* means the number of tests with cohesive fracture in the substrate.

4. Comparison of results from the EAD/EAD′ and CAST methods;

For selected mortars, a comparison of mean bond strength obtained using both methods was performed, as illustrated in Figure 12. The red bar in each chart represents results from the EAD-compliant method [15], the next two bars represent results from the EAD-based method (EAD′), and the remaining bars show CAST results under eight experimental conditions (c1–c8) specified in Table 1. Six mortars were selected, for which the results reflect the possible variability of mean bond strength under different adhesive mortar application conditions.

For half of the six representative adhesives, EAD (including EAD′) yields highly comparable bond-strength levels across the three substrates, which may indicate that at this scale the method is only slightly sensitive to substrate-specific behavior. In contrast, CAST appears more discriminative: it consistently amplifies material- and use-case–specific responses (substrate × orientation × thickness), revealing patterns that remain muted under EAD. In particular, CAST highlights (i) the strong thickness penalty on concrete, (ii) the milder orientation penalty on silicate, and (iii) notable substrate–thickness interactions that vary by adhesive.

These contrasts should be read with the available sample sizes in mind (some cells have few results), but the qualitative pattern is consistent across all six examples.

Figure 12a displays the comparative results for adhesive a1. In CAST, on the same concrete slab and in the horizontal orientation, using a 10 mm adhesive layer—still considered acceptable in practical ETICS applications—the mean bond strength is 75% higher than in EAD′. On the silicate substrate, CAST reveals markedly lower values across virtually all test conditions, in several cases leading to premature specimen detachment before testing (*n* < 5). By contrast, under EAD′ on the same substrate, the mean level remains very high.

Figure 12b summarizes the data for adhesive a7. Under EAD, the values are relatively low yet meet the required minimum. In EAD′ on the concrete slab, the mean level is higher; however, this does not carry over to CAST on the same substrate. In CAST on the concrete slab, the vertical configuration with a 10 mm adhesive layer yields a mean bond strength about 69% lower than the horizontal configuration at the same thickness. By contrast, on the silicate block, CAST yields very high mean bond strength values, with cohesive fracture in the substrate (CFS) observed; nevertheless, differences between horizontal and vertical orientations are noticeable.

Figure 12c depicts the findings for adhesive a8. This case is somewhat specific because in CAST on the concrete slab, the horizontal 10 mm configuration exhibits very high mean bond strength, whereas switching to the vertical orientation results in an almost tenfold reduction. Increasing the adhesive layer to 20 mm further decreases the mean, yet it remains above the EAD requirement [15]. On the silicate block, the 10 mm layer yields comparatively high means—particularly in the horizontal orientation—while the vertical orientation lowers them. With 20 mm, a further decrease is observed and the horizontal–vertical gap narrows; the 95% BCa confidence intervals are relatively wide and partly overlapping. Meanwhile, in EAD/EAD′, the bond strengths on concrete are comparable and slightly lower than on silicate; nevertheless, all EAD/EAD′ values lie below the CAST result on silicate in the horizontal 10 mm configuration.

Figure 12d presents the results for adhesive a9. This is another specific case, characterized by high and nearly uniform means in EAD/EAD′ across all substrates. In CAST on the concrete slab, the horizontal 10 mm configuration attains a very high mean, whereas the vertical 10 mm configuration is clearly lower—by about 59%—yet still high. On the silicate block, CAST reveals very low bond strength in all thickness–orientation settings, including near-zero means and incomplete series (*n* < 5) due to specimen detachment.

Figure 12e shows the outcomes for adhesive a10. A different picture emerges: although EAD/EAD′ is moderate, CAST delivers very high mean bond strength on both substrates. The horizontal 10 mm configuration is the strongest; increasing the thickness and switching to the vertical orientation follow a similar downward trend, yet even the lowest CAST means remain comparatively high. On silicate, CFS is observed, consistent with substrate-limited failure.

Figure 12f reports the measurements for adhesive a15. In this case, EAD/EAD′ yields comparable, relatively low means, and CAST largely mirrors this behavior: most configurations remain low, with a single favorable setting—concrete, horizontal, 10 mm—reaching a high value. Increasing thickness frequently coincides with specimen loss: on concrete, the vertical 20 mm series yields *n* = 3, while on silicate, both 20 mm series return *n* = 0 (detachment during conditioning), indicating very low bond.

In the interpretation of the test results obtained with the CAST method, it is important to recall that the element for which adhesion is determined is pulled off from the central part of the adhesive dab, that is, from the area with potentially the highest adhesion to the substrate. In cases where the central element of the test specimen shows relatively low adhesion to the substrate, its remaining parts simply detach already during the cutting of the specimen. This demonstrates a decrease in adhesion values toward the outer areas of the dab. Another important issue concerns the fact that the specimen, formed as an adhesive application, has the same shape and size as the dab used in actual thermal insulation bonding. This also applies to the thickness of the adhesive layer. This, in turn, implies certain behaviors of the mortar itself in relation to the substrate, such as its wetting, water absorption from the mortar, pressure comparable to that in real applications, possible shrinkage during curing and its effect on bonding forces with the substrate. The position of the specimen, which is also related to the influence of gravity on the physical and chemical processes occurring between the mortar and the substrate, is not without significance for the results.

The analysis of the failure patterns of all samples tested using the CAST method leads to the conclusion that, in most cases, adhesive failure occurs at the interface between the substrate and the cement-based adhesive mortar. This is a well-known and widely described phenomenon in research and literature, referred to as the interfacial transition zone (ITZ), as discussed, among others, in study [49]. Thus, we are dealing with a certain weakening of the microstructure within the very thin layer of mortar in comparison to the mortar core, where cement hydration proceeds differently due to the varying amount of available water. In this area, increased mortar porosity also occurs, which weakens the bonding process of the cement binder.

At the contact with the substrate, chemical reactions dependent on both the properties of the mortar and the type of substrate (chemical adhesion) may also take place. Furthermore, effects of mechanical interlocking were observed, depending on the porosity of individual substrates (mechanical adhesion). The spectrum of different types of adhesion, along with their characteristic features, is also described in research work [50], with references to additional literature sources.

The observed fracture patterns in the samples tested using the CAST method indicate analogies with the definitions of adhesion types. The varied tensile bond strength results of individual mortars to different substrates confirm their distinctive characteristics resulting from their specific formulations. These, in turn, cause greater or lesser sensitivity to certain substrate properties and influence the formation of the ITZ structure, which affects the mortar’s resistance to debonding from the substrate.

According to research studies [51,52], the improvement of adhesion in cement-based adhesive mortars is influenced by polymer compounds, specifically redispersible polymer powders. The polymer film “seals” the pores in the ITZ, partially preventing water penetration and salt migration, which also contributes to the durability of adhesion—particularly under moisture exposure and freeze–thaw cycles. The influence of polymers on the cement phase results in a “flexibilization” of the system, increasing deformation capacity under tensile stress. The presence of polymers can also enhance the penetration of mortar particles into the micro-pores of the substrate and reduce shrinkage during setting.

#### 3.3.2. Inferential Statistics

1. CAST factorial (substrate × orientation × thickness)

Across the CAST datasets, the thickness factor (10 vs. 20 mm) typically dominated the variance structure. PERMANOVA assigned large shares of explained variance to thickness in most adhesives, with R^2^ typically in the ~0.25–0.88 range (e.g., a2, a3, a4, a10, a12, a14), and permutation ANOVA yielded commensurately large *η*^2^. In several cases (e.g., a1, a8, a9), the thickness effect approached the lower bound of this range, whereas in a few adhesives (e.g., a6, a7, a11) it remained small and was overshadowed by other factors or interactions.

Orientation (horizontal vs. vertical) contributed an independent, practically important effect, with PERMANOVA R^2^ commonly ~0.12–0.32 (particularly large in a8), and its impact was frequently magnified at 20 mm, as reflected by a significant o × t interaction (observed, e.g., in a1, a8, a9, a11, a15; R^2^ up to ~0.29). The substrate factor was adhesive-dependent: R^2^ ranged from ~0.02 to ~0.84 (notably high in a7, and elevated in a1, a5, a6, a9, a13, a14), but was often secondary to interactions—especially s × t (up to ~0.25; a1, a4, a5, a6, a13) and s × o (up to ~0.24; a11)—indicating that both the thickness penalty and the horizontal–vertical gap are substrate-specific. Three-way effects (s × o × t) appeared less frequently (e.g., a1, a3, a5, a8, a11) and with small shares of variance (R^2^ generally ~0.01–0.07), but they reinforce the presence of non-additivity in a subset of adhesives. These conclusions were robust under the aligned rank transform (ART), which reproduced the pattern of significant main effects and the key interactions.

Pairwise, Holm-adjusted Wilcoxon contrasts localized the dominant differences. The most recurrent signals were t2 < t1 within each (s,o) cell and horizontal > vertical within each (s,t) cell. The corresponding effect magnitudes were substantial: Cliff’s *δ* typically |*δ*| ≈ 0.35–0.90 (occasionally approaching 1.00) and Vargha–Delaney A (VD-A) often lay in the 0.10–0.30 (or 0.70–0.90) range on the strongest contrasts (e.g., horizontal vs. vertical at 10 mm, or 10 vs. 20 mm within vertical). Substrate contrasts were more adhesive-specific and interaction-dependent, with some adhesives (e.g., a6, a7, a9, a13, a14, a15) showing clearly dominant substrates, while others displayed more balanced patterns.

As an omnibus consistency check, one-way rank tests across the eight CAST combinations (c1–c8) were consistently significant, with global Kruskal–Wallis epsilon-squared *ε*^2^ typically ~0.72–0.90 (median ≈ 0.86), corroborating the factorial breakdown and aligning closely with the CAST one-way analyses.

Taken together, CAST shows that reducing thickness and avoiding the vertical-orientation penalty are the most impactful levers for bond strength, whereas the substrate modulates the size of these effects in an adhesive-dependent manner. Statistically, this picture is supported by large R^2^/*η*^2^, non-trivial *δ*/VD-A, and robust agreement with ART across the dataset.

2. CAST one way by conditions (c1–c8)

Treating the eight CAST combinations (c1–c8) as a single eight-level factor yielded consistently strong omnibus signals across adhesives. Kruskal–Wallis tests were significant for all datasets (*p* < 0.05), with epsilon-squared *ε*^2^ spanning ~0.70–0.90 (median ≈ 0.86), indicating large differences across the eight conditions. In a few cases (e.g., a11, a15) *ε*^2^ values were closer to the lower bound of this range (~0.70), but still well within the “large” category by conventional thresholds. In parallel, the one-way PERMANOVA assigned large shares of explained variance to the eight-level factor, with R^2^ spanning ~0.68–0.98 (median ≈ 0.93), and permutation ANOVA produced *η*^2^ of comparable magnitude. These omnibus results are fully aligned with the factorial breakdown, confirming that CAST levels combine into well-separated groups when collapsed to (c1–c8).

Holm-adjusted pairwise Wilcoxon comparisons revealed the expected pattern of contrasts, albeit with conservative multiplicity control. While adjusted *p*-values were sparse, effect sizes were overwhelmingly large: Cliff’s *δ* magnitudes were predominantly in the large range (the vast majority of pairwise contrasts), with a median |*δ*| ≈ 1.00 (IQR ≈ 0.84–1.00). Corresponding Vargha–Delaney A showed a polarized distribution consistent with strong separation among c1–c8. Taken together, the one-way analysis corroborates the factorial conclusion that differences among CAST conditions are substantial in magnitude, even when pairwise significance is attenuated by Holm correction.

In practical terms, the one-way view reinforces that horizontal 10 mm configurations tend to anchor the upper tail of performance, whereas vertical and/or 20 mm settings populate the lower tail; the precise ranking among c1–c8 is adhesive-dependent, matching the interaction structure seen in the factorial analyses. Consistently, the ranking inferred from pairwise effect sizes mirrors the visual ordering in Figure 12 (panels a–f): c1 (s1-o1-t1) typically belongs to the upper tier, c3 (s1-o2-t1) sits lower than its horizontal counterpart, c5 (s2-o1-t1) exceeds c7 (s2-o2-t1), and the t2 variants are shifted downward; deviations are adhesive-specific and reflect the s × o × t interaction.

3. EAD one-way by substrate (s0–s2)

Treating substrate as a three-level factor yielded a mixed but interpretable pattern across adhesives. Roughly half of the datasets showed statistically significant omnibus differences among substrates, with large effect sizes when significant. Specifically, Kruskal–Wallis *ε*^2^ values in these cases typically ranged from ~0.50 to 0.74 (e.g., a1, a5, a6, a8, a10, a11, a13, a14), confirming substantial dispersion across substrates. In other adhesives (e.g., a3, a4, a9, a12, a15), *ε*^2^ was close to zero, indicating negligible substrate-related variance. PERMANOVA results were fully consistent with this picture, assigning very large shares of variance to substrate when significant (R^2^ ≈ 0.47–0.90) and very small shares when not (R^2^ ≈ 0.04–0.18). Permutation ANOVA produced *η*^2^ values of comparable magnitude, reinforcing the robustness of the omnibus results.

Pairwise Wilcoxon contrasts (Holm-adjusted) clarified the direction of differences where present. Several adhesives placed the third substrate at the top of the ranking with very large pairwise effects (e.g., a6, a8; Cliff’s *δ* ≈ −1 in comparisons vs. s0), while others favored the first substrate (e.g., a10, a11; *δ* ≈ +1 vs. s1). A few cases (e.g., a5) suggested a second/third > first pattern with large *δ*, though adjusted *p*-values sometimes hovered near the 0.05 threshold, underscoring that effect magnitude can remain large even when family-wise error control suppresses significance. Conversely, adhesives with non-significant omnibus results showed pairwise effects that were small or highly uncertain, reflecting the negligible overall variance partitioning in those cases.

Putting these results together, the EAD one-way analysis indicates that (i) marked substrate differentiation occurs in multiple adhesives with large omnibus effect sizes (*ε*^2^ and PERMANOVA R^2^), (ii) the identity of the best-performing substrate is adhesive-dependent, and (iii) in the remaining adhesives, substrate differences are statistically small and practically modest. This substrate-specificity under EAD dovetails with the CAST factorial picture, where substrate effects are frequently modulated by orientation and thickness. In other words, the EAD substrate ranking provides a coherent baseline that is selectively amplified or reversed once orientation and adhesive-layer thickness are introduced in CAST.

## 4. Conclusions

The thickness of the adhesive layer for thermal insulation in actual ETICS applications is significantly greater than the thicknesses recommended by the manufacturers of the tested adhesive mortars. The most frequently occurring adhesive layer thicknesses fall within the range of 10–25 mm. Most contractors in Poland also declare that the most commonly encountered maximum adhesive layer thickness exceeds 20 mm.

The adhesion of cement-based adhesive mortars to the substrate depends on the thickness of the adhesive layer, the position of specimen bonding, the type of substrate and the properties of the adhesive mortar. The thickness of the adhesive layer also implies a relationship with the pressure exerted on the mortar and the substrate.

The results obtained using the CAST method differ significantly from those of the EAD and EAD’-based methods. This conclusion concerns tests carried out under laboratory (“dry”) conditions. This means that some mortars achieving relatively high results in the EAD method for specific substrates may not achieve significant adhesion in the CAST method, particularly in vertical exposure.

Most mortars tested by the CAST method in the variant with specimens bonded and cured in the vertical position showed significantly lower adhesion to both types of substrates. In some cases, the differences are very large.

An increase in the adhesive layer thickness from 10 mm to 20 mm on concrete substrates reduces bond strength by approximately 65–75%. A vertical orientation decreases adhesion to the substrate by about 50% compared to the strength obtained in a horizontal position. Therefore, a change in adhesive layer thickness has a greater impact on reducing tensile bond strength than a change in the substrate’s orientation (position). The combined effect of increased thickness and vertical orientation on concrete substrates reduced adhesion by about 85% compared to samples with a 10 mm thickness and horizontal orientation.

In the case of calcium silicate block substrates, the effects were weaker but more variable. When using averages from a subset limited to ratios not exceeding 1, an increase in adhesive thickness from 10 mm to 20 mm resulted in a decrease in the adhesion of thermal insulation to the substrate by approximately 41–51% (≈51% in horizontal orientation, ≈41% in vertical orientation). A change in orientation from horizontal to vertical reduced adhesion by about 42% for the 10 mm layer and by 31% for the 20 mm layer. The combined effect reduced the average adhesion by approximately 55%.

The aim of this research work was to determine the actual thicknesses of adhesive layers most commonly used when bonding thermal insulation in ETICS, and to highlight the significance of the properties of adhesive mortars as they manifest in real applications. Consequently, the study also aimed to initiate a discussion on improving the testing and evaluation process of adhesive mortars in terms of all characteristics that are essential for effective bonding.

The comparison of results obtained using both methods made it possible to gain new knowledge in the subject area. This will contribute to improving bonding effectiveness through a deeper understanding of the mechanisms occurring at the adhesive–substrate interface.

The primary objective is to adapt testing methods to real installation conditions of thermal insulation systems, particularly those related to the thickness of the adhesive layer and the type of substrate.

The results of the conducted research may contribute to a significant improvement in the effectiveness of adhesive bonding of thermal insulation applied with increased adhesive layer thickness, which, as the analyses show, is commonly used in practice. New criteria for evaluating the adhesion of mortars may help minimize the risk of their excessive sensitivity to the varying properties of different substrates. Consequently, this new approach will enhance the operational durability of thermal insulation systems and improve user safety, which has both economic and social implications.

The environmental benefit lies in the fact that the insulation will continue to reduce heat loss for a longer period, maintaining low energy consumption for heating purposes. Extending the service life of the insulation will also positively affect the carbon footprint over the building’s life cycle.

Further research recommendations by the authors include a broader validation of the CAST method, interpretation of the obtained results, and gathering feedback from other researchers regarding the applicability of this tool as a complementary method to the EAD procedure under laboratory conditions.

Additional recommended research areas include studies on the influence of substances that modify substrate properties—such as priming, coating, and penetrating agents—on the tensile bond strength results, as well as determining the effects of extreme application conditions and operating temperatures on the effectiveness of the bond between the adhesive, substrate, and thermal insulation (EPS). Moreover, future studies could consider conducting CAST with the inclusion of freeze–thaw cycles. However, due to the potential variability in wall substrate types (e.g., calcium silicate blocks, ceramic units, others) and the size of test samples, this may represent a significant limitation of the method. Therefore, the authors currently recommend its application primarily under laboratory conditions. Ultimately, the most important objective is to determine the maximum and safe adhesive layer thickness in ETICS, which can be established using optimized evaluation methods.

## Figures and Tables

**Table 1:** Coding of experimental conditions (c1–c8) by substrate (s1, s2), orientation (o1, o2), and adhesive thickness (t1, t2). Cells are merged where conditions share the same substrate or orientation.

Condition Codes	Substrate	Substrate Orientation	Adhesive Thickness [mm]
c1: s1-o1-t1	Concrete slab	Horizontal	10
c2: s1-o1-t2			20
c3: s1-o2-t1		Vertical	10
c4: s1-o2-t2			20
c5: s2-o1-t1	Silicate block	Horizontal	10
c6: s2-o1-t2			20
c7: s2-o2-t1		Vertical	10
c8: s2-o2-t2			20

**Table 2:** Minimum, maximum, and median bond strength values for the CAST method under conditions c1–c8.

Condition Codes	Min [MPa]	Max [MPa]	Median [MPa]
c1: s1-o1-t1	0.1696	1.8576	0.7218
c2: s1-o1-t2	0.0390	0.6968	0.1718
c3: s1-o2-t1	0.0532	1.3968	0.3628
c4: s1-o2-t2	0.0340	0.3868	0.1176
c5: s2-o1-t1	0.0310	1.3754	0.1420
c6: s2-o1-t2	0.026 (0.0335)	1.1102	0.2570 (0.3048)
c7: s2-o2-t1	0.0182	1.2744	0.2688
c8: s2-o2-t2	0.035 (0.0370)	0.9208	0.2034 (0.2483)

Note: Values in parentheses indicate recalculated statistics after excluding single-measurement cases that could not be considered as averages.

**Table 3:** Mean and median bond strength ratios (dimensionless) between mortar layers of 20 mm and 10 mm thickness (t2/t1), with 95% bootstrap confidence intervals, CI.

Substrate	Orientation	Mean [95% CI]		Median [95% CI]	
		All Results	Results ≤ 1	All Results	Results ≤ 1
Concrete slab	Horizontal	0.3334 [0.232, 0.627]	0.2545 [0.198, 0.321]	0.2571 [0.148, 0.334]	0.2266 [0.148, 0.332]
	Vertical	0.3432 [0.234, 0.638]	0.2583 [0.202, 0.339]	0.2629 [0.148, 0.320]	0.2488 [0.142, 0.311]
Silicate block	Horizontal	1.2840 [0.656, 2.629]	0.4917 [0.339, 0.653]	0.6723 [0.321, 1.515]	0.4444 [0.236, 0.707]
	Vertical	1.0639 [0.648, 2.427]	0.5940 [0.431, 0.716]	0.7517 [0.479, 0.890]	0.6852 [0.309, 0.758]

**Table 4:** Mean and median bond strength ratios (dimensionless) between vertical and horizontal substrate orientations (o2/o1), with 95% bootstrap confidence intervals, CI.

Substrate	Adhesive Thickness	Mean [95% CI]		Median [95% CI]	
		All Results	Results ≤ 1	All Results	Results ≤ 1
Concrete slab	10 mm	0.5653 [0.432, 0.710]	0.5267 [0.398, 0.652]	0.5245 [0.363, 0.781]	0.4824 [0.314, 0.631]
	20 mm	0.5321 [0.410, 0.688]	0.4848 [0.375, 0.602]	0.4911 [0.252, 0.605]	0.4713 [0.252, 0.605]
Silicate block	10 mm	0.9438 [0.566, 2.269]	0.5792 [0.458, 0.709]	0.5871 [0.392, 0.838]	0.5709 [0.337, 0.750]
	20 mm	0.9107 [0.701, 1.362]	0.6896 [0.544, 0.824]	0.8238 [0.550, 1.026]	0.6921 [0.429, 0.873]

Note: Identical CI values in some conditions are a result of the BCa bootstrap method and do not indicate a calculation error.

**Table 5:** Mean and median bond strength ratios (dimensionless) for c4/c1 (concrete slab) and c8/c5 (silicate block), with 95% bootstrap confidence intervals, CI.

Substrate	Conditions Ratio	Mean [95% CI]		Median [95% CI]	
		All Results	Results ≤ 1	All Results	Results ≤ 1
Concrete slab	c4/c1	0.1449 [0.111, 0.188]	No ratios exceed 1	0.1265 [0.069, 0.158]	No ratios exceed 1
Silicate block	c8/c5	0.9310 [0.459, 1.867]	0.4517 [0.330, 0.579]	0.5376 [0.261, 0.706]	0.4461 [0.243, 0.634]

## Data Availability

The original contributions presented in this study are included in the article. Further inquiries can be directed to the corresponding authors.

## Abbreviations

The following abbreviations are used in this manuscript:

ETICS	External Thermal Insulation Composite Systems
EAD	European Assessment Document; also used to denote the pull-off testing method defined therein
EAD′	Variant of the EAD method applied to “non-normative” substrates
EPS	Expanded Polystyrene
TR	Tensile Strength Perpendicular to Face (EN 1607)
MPa	Megapascal
CAST	Central Adhesive Spot Test (in-house method)
CFS	Cohesive Fracture in Substrate
LE1	Results limited to values ≤ 1 (Low, Equal 1)
CI	Confidence Interval
BCa	Bias-Corrected and Accelerated (bootstrap CI)
SW	Shapiro–Wilk (test)
KS	Kolmogorov–Smirnov (test)
CvM	Cramér–von Mises (test)
AD	Anderson–Darling (test)
AIC	Akaike Information Criterion
BIC	Bayesian Information Criterion
Q–Q	Quantile–Quantile
MLE	Maximum Likelihood Estimation
SD	Standard Deviation
SE	Standard Error
IQR	Interquartile Range
MAD	Median Absolute Deviation
MedAD	Mean Absolute Deviation from the Median
MedAD_n_	Scaled Median Absolute Deviation (normalized)
PEMANOVA	Permutational Multivariate Analysis of Variance
ANOVA	Analysis of Variance
R^2^	Coefficient of Determination (as variance explained)
*η*^2^	Eta-squared (effect size)
*ε*^2^	Epsilon-squared (effect size)
*δ*	Cliff’s delta (effect size)
VD-A	Vargha–Delaney A (effect size)
ART	Aligned Rank Transform
*ρ*	Spearman’s rank correlation coefficient
*τ*	Kendall’s rank correlation coefficient
*p*	Probability value (statistical significance)
GenAI	Generative Artificial Intelligence
IDE	Integrated Development Environment
